# Supplementary material for: Mechanisms of cultural diversity in urban populations
Source: Nat Commun. 2025 Jun 4;16:5192. doi: 10.1038/s41467-025-60538-2 (PMC12137573; doi:10.1038/s41467-025-60538-2)
Supplement: Supplementary file 1 — Supplementary Information [file 41467_2025_60538_MOESM1_ESM.pdf]

Supplementary information

Mechanisms of Cultural Diversity in Urban Populations

Harin Lee[1,2,3,4,✳], Nori Jacoby[3,5], Romain Hennequin[1,✢], Manuel Moussallam[1,✢]

[1] Deezer Research, Paris, France
[2] Max Planck Institute for Human Cognitive and Brain Sciences, Leipzig, Germany
[3] Max Planck Institute for Empirical Aesthetics, Frankfurt am Main, Germany
[4] Department of Life Sciences, Leipzig University, Leipzig, Germany
[5] Department of Psychology, Cornell University, Ithaca, US

[✳] hlee@cbs.mpg.de
[✢] These authors jointly supervised this work

All scripts for the analyses and data are openly available at:
https://github.com/harin-git/mus-div

Supplementary Text

Note 1: Causal inference step-by-step procedure.

Our study employs a structured causal inference approach to explore the impacts of demographic and social factors on between and within-level diversity outcomes. Here, we describe the full step-by-step procedure of causal inference testing we applied, which generally adopts the protocol proposed by Ankan et al.[1].

1. Constructing the DAG

Using Directed Acyclic Graphs (DAGs), we modelled the hypothesised causal relationships among variables that guide our analysis. Potential confounders were chosen based on existing literature and they are detailed in the ‘Socio-demographics’ section of the main text. DAG provides a clear and efficient method to identify, present, and hypothesise the causal relationships between variables. Our main aim in illustrating the model with a DAG is to transparently lay out our assumptions that can be refuted and corroborated by future researchers to serve as a useful framework.

2. Assumptions and sanity checks

Given that the causal testing we perform assumes normality, we performed log transformation for variables that did not follow a normal distribution as assessed by Q-Q plots (see Supplementary Table 5 for the distribution of all variables). Next, prior to model fitting, we assessed potential collinearity among the variables through covariance and correlational analyses. No pairwise correlations were larger than 0.92 and no variable was a linear combination of two variables.

3. *Model specification*

We defined two models for testing causal effects, as illustrated in Figures 3a,b in the main text:

Model 1: Effects of population size on between-individual diversity (BID)
Model 2: Effects of population size on within-individual diversity (WID)

For each model, we first tested the implied conditional independence of our DAG using the *localTests* function using the R package *dagitty*[2]. Implied conditional independence is a method used to verify whether the assumptions of conditional independence hold true in a given probabilistic graphical model. In other words, it checks if some variables in the model are indeed independent of others, given the values of some other set of variables. The test is important because it helps ensure that the model accurately represents the relationships and dependencies

among the variables. If the test fails (i.e., large effects are observed between variables that are not included as links in the model), it implies the model may need to be adjusted to more accurately reflect the true relationships among the variables. On our DAG, the test revealed no substantially large effects, suggesting the relationship we assume is not refuted by the data.

Next, we identified the minimal adjustment sets (i.e., variables to include as confounders). Minimal adjustment sets are the smallest sets of variables that, when controlled, can eliminate confounding bias in causal inference studies[3]. Identifying specific confounders to control, rather than controlling for all possible confounders (i.e., throwing everything into the sink), is important to avoid misleading conclusions that can arise from relationships between the variables, such as the 'collider bias'[4].

4. Propensity score weighting

The minimal adjustment sets identified for each model in the previous section were controlled using the propensity score weighting method[5]. This method involves estimating the probability of treatment assignment (in our case, the size of the area they live), known as the propensity score, for each individual based on their observed covariates. In a randomised experiment, this treatment probability is known, but in observational studies, it needs to be estimated, typically using logistic regression.

Once the propensity score is estimated, it is used to weigh each individual by the inverse of the probability of receiving the treatment they actually received. If they did not receive the treatment, they are weighted by the inverse of one minus their propensity score. This process synthesises a sample in which the distribution of observed covariates is independent of treatment assignment. In essence, this method summarises all covariate information into a single score for each participant, which is then used to create a balanced and unbiased sample for estimating the causal effect of the treatment. This mimics the scenario of a randomised controlled trial, thereby reducing the bias due to confounding variables, and allowing for more accurate estimates of causal effects in observational studies.

We applied the Average Treatment for the Overlap population (ATO) weighting procedure using the R package *WeightIt*[6]. Unlike traditional propensity score methods such as ATE or ATT, ATO weights mitigate issues of extreme weight variance as they are inherently bounded between 0 and 1, thus avoiding the need for trimming or winsorising (for issues related to weight trimming, see Lee et al.[7]). This was supported by our diagnostic checks which showed that our model achieves good covariate balance, demonstrated through standardised mean differences and empirical cumulative distribution function analyses (Supplementary Figs. 14 and 15).

The obtained propensity scores for each individual were then used as weights in a linear model when predicting the outcomes of BID and WID as a function of population size.

5. Error estimation

To assess the robustness of our models, we used bootstrap sampling with replacement to obtain confidence estimates. We ran 1,000 simulations each with newly sampled sets of individuals and by reapplying the entire weight assignment procedure.

Supplementary Figures

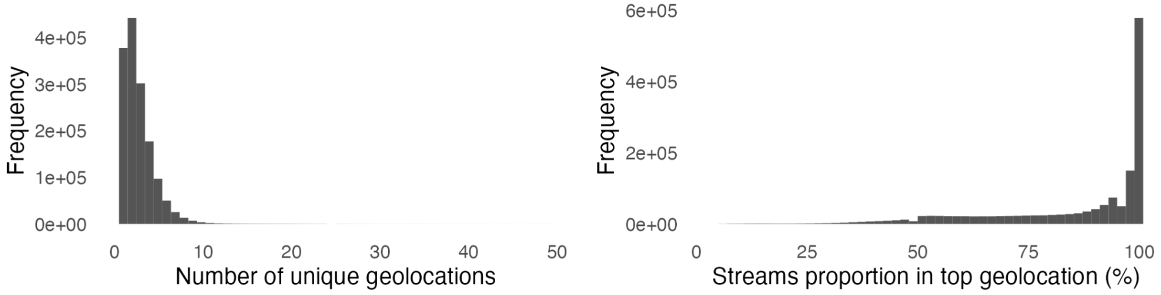

Fig. 1: User geolocations.

Histogram of the number of distinct geo locations identified for each user within the French sample (left), and the percentage of streams originating from the most frequent geolocations of each user (right).

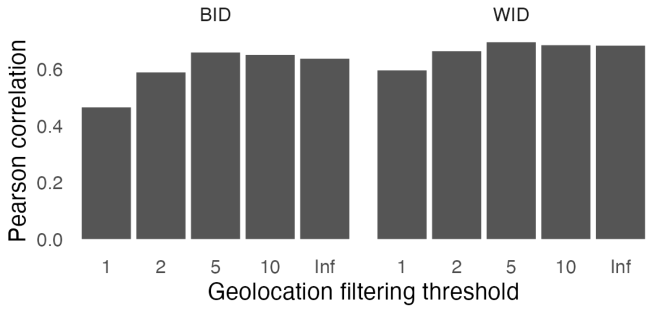

Fig. 2: Varying user geolocation filtering criteria.

Applying stricter or looser criteria for geolocation user filtering by varying the number of unique locations a user can have to be included in the sample. The Y-axis represents two-tailed Pearson correlations with population size (log base of 10), suggesting a robust positive correlation even when applying the strictest criteria (i.e., including only users that stream from a single location). Main study uses 10 as the threshold.

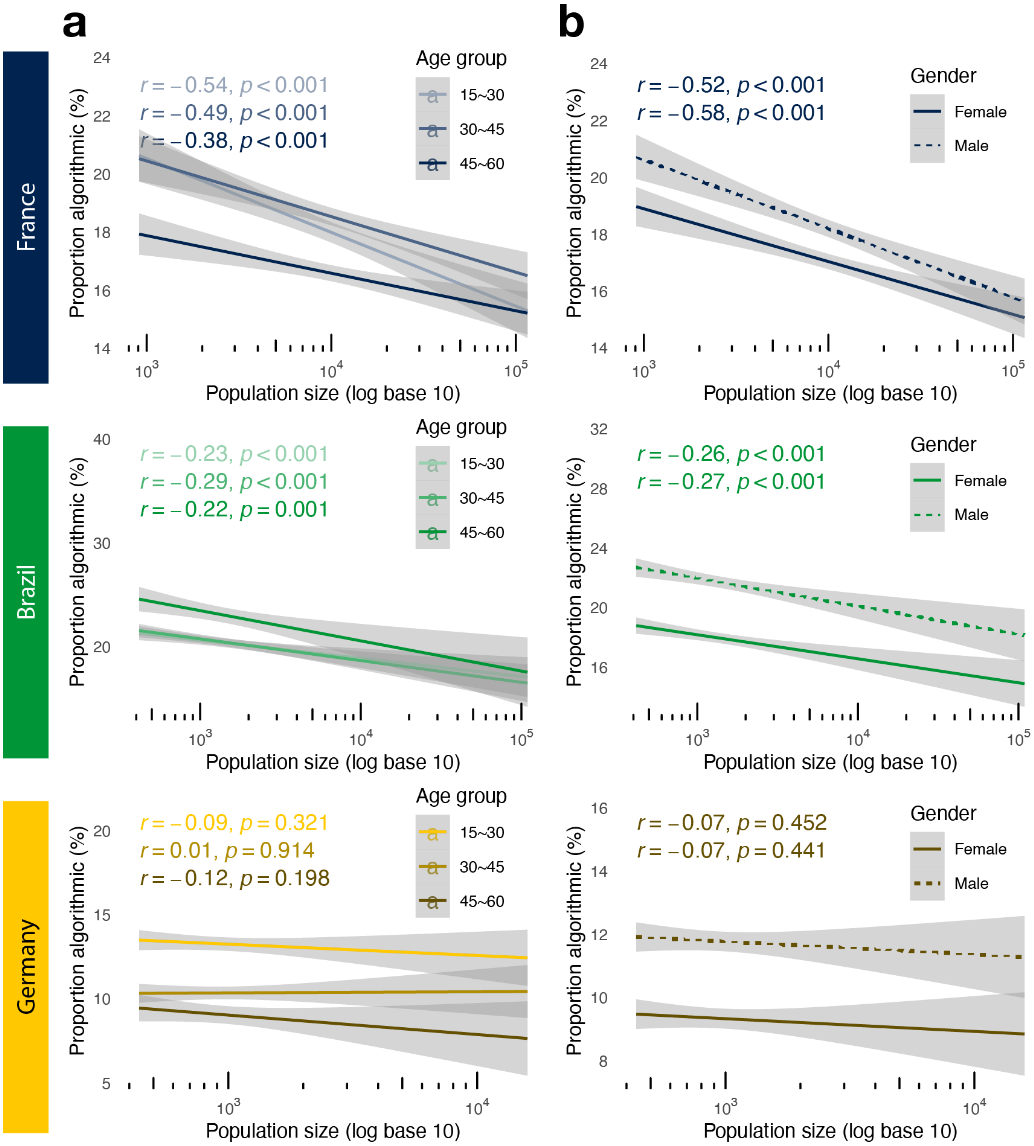

Fig. 3: Usage of algorithmic recommendations.

(a) Algorithmic recommendation proportion as a function of population size for different age groups. There is a negative two-tailed Pearson correlation in France and Brazil, indicating that users in large metropolitan areas use recommendations less, regardless of age. However, this effect is not present in Germany. **(b)** Algorithmic recommendation proportion as a function of population size comparing male and female users. There is a group-level difference across all three countries where male users tend to use around 2% more algorithmic recommendations than Female users. GLM is fitted across all analyses with shaded areas indicating 95% CI. Correlations were not corrected for multiple comparisons.

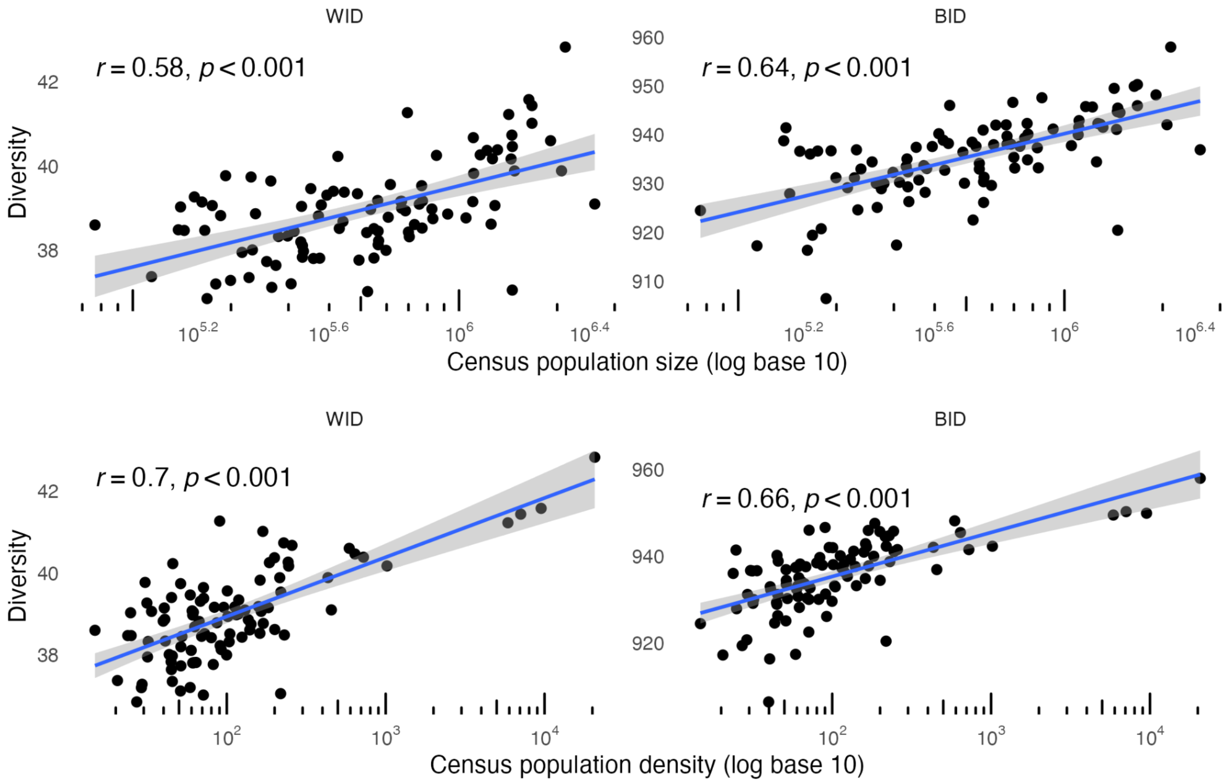

Fig. 4: Census population size and population density.

Relationships between BID and WID with Eurostat census data on population size and population density (log base 10) in France (NUT3 unit regions, N = 96). GLM is fitted with shaded areas representing 95% CI. Two-tailed Pearson correlation is computed without correcting for multiple comparisons.

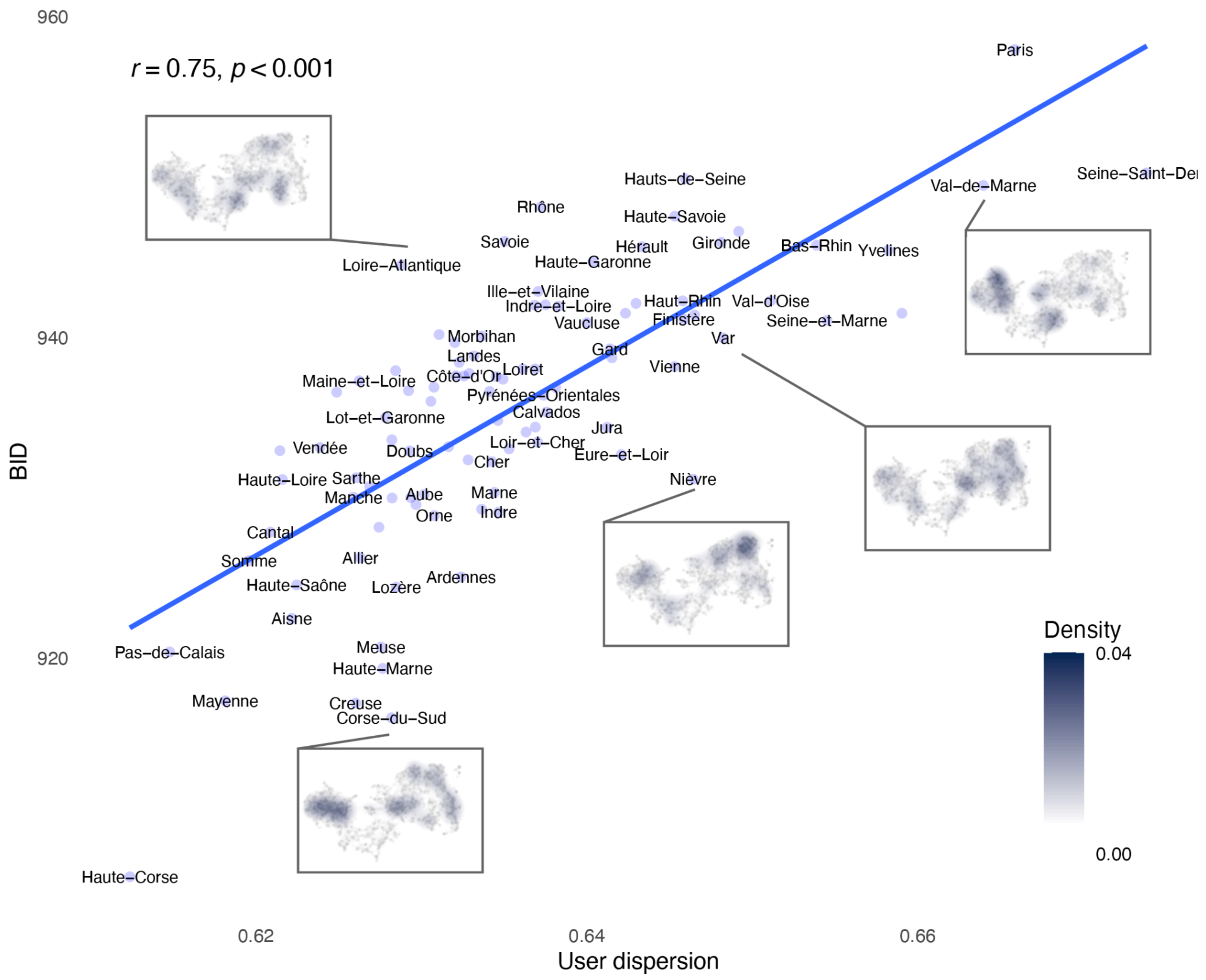

Fig. 5: BID and user dispersion.

Extended version of Figure 1d in the main text comparing the BID measure with user dispersion. User dispersion is computed based on the cosine distance across all pairs of sampled individuals in a given area (i.e. full matrix; NUTS3 unit regions in France, N = 96), and then computing population variance (‘Dispersion in user embedding’ section in Methods). The strong positive two-tailed Pearson correlation validates the robustness of our BID measure. Note the analysis could not be run for Brazil and Germany as the raw user embeddings for these countries were not extracted during the data collection phase.

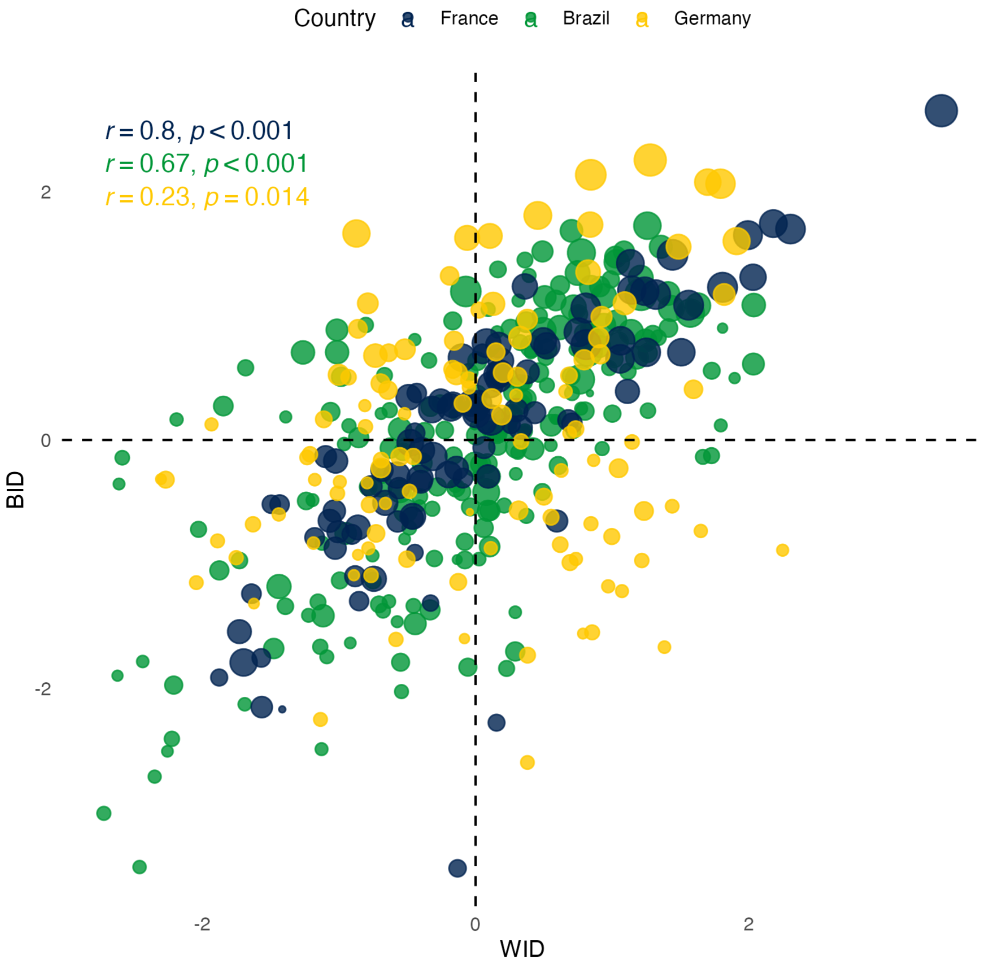

Fig. 6: Correlation between WID and BID.

Relationship between WID and BID. Each dot is coloured by the country and its size is proportional to the population size of the area (N geographical areas: France = 96, Germany = 113, and Brazil = 218). All values are obtained from bootstrapped means and are normalised using Z-scores by country. Two-tailed Pearson correlation is computed without correcting for multiple comparisons. Interestingly, Germany demonstrates a relatively smaller correlation between WID and BID, suggesting that areas where an individual's cultural breadth is large do not necessarily strongly correspond to them also having more distinct music tastes from one another.

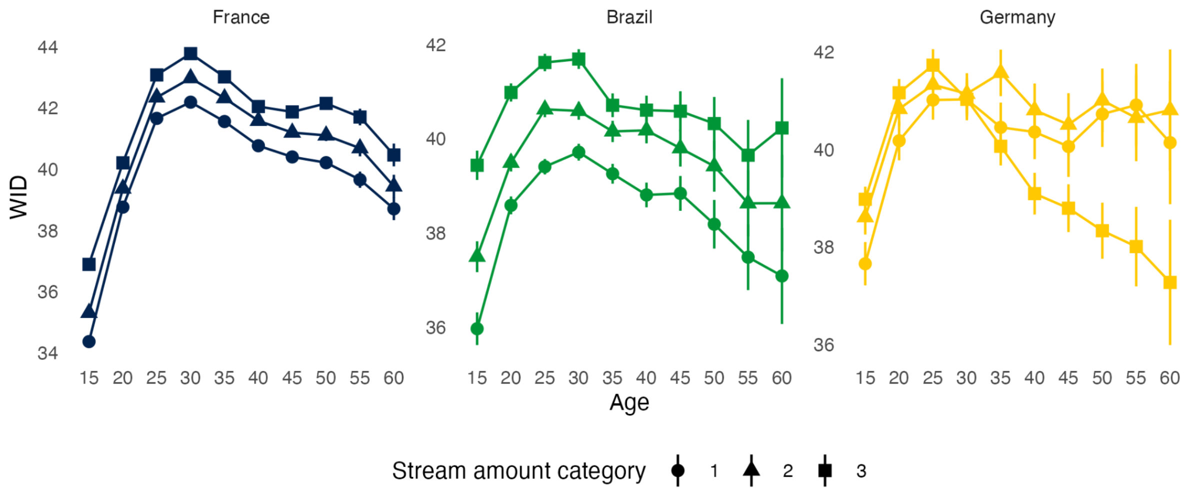

Fig. 7: Controlling for the number of streaming activities.

WID as a function of age, stratified by streaming intensity categories, showing consistent inverted U-shape trajectories. A user's stream amount is divided into three quantile groups (1 being the least and 3 being the most) by the number of streams within the 28-day period. Age is grouped by a five-year window. Each point represents the group mean and error bars represent 95% CI of the mean.

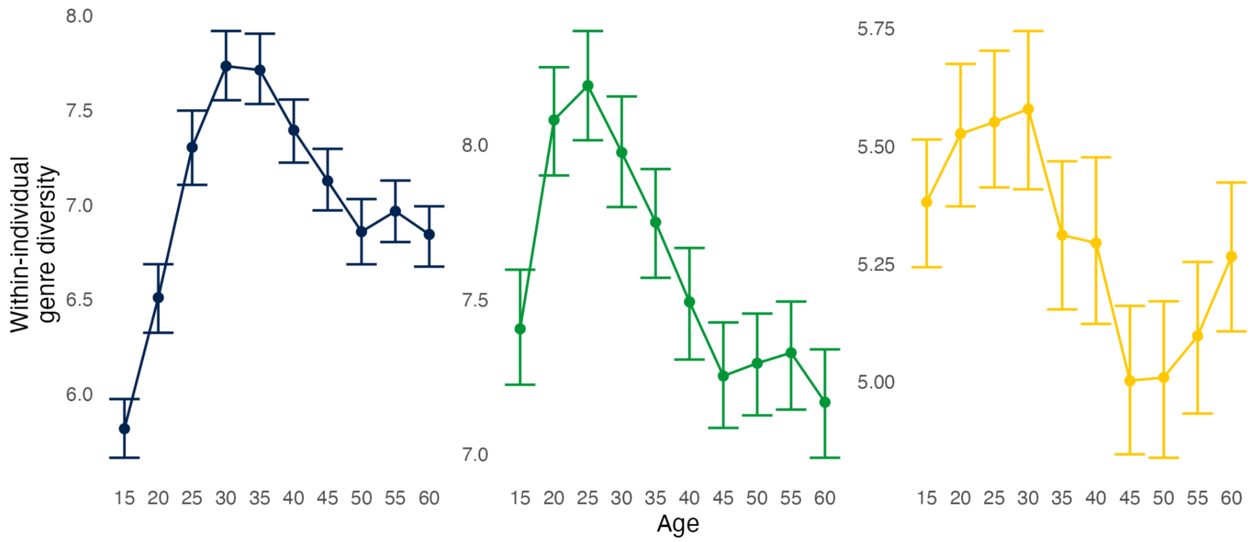

Fig. 8: Within-individual genre diversity.

Within-individual level genre diversity as a function of age. Genre diversity per individual was computed based on the frequency of genre tags for the 100 streams sampled per user. Hill's number of order $q = 1$ was used for consistency with the BID measure ('Between-individual diversity (BID)' section of the main text). When the song contained multiple genre tags, the primary (the first) label was used. For each age category in bins of five years, 1,000 users were drawn at random, resulting in a total of 10,000 user samples per country. Among these sub-samples, bootstrap simulation was performed to compute 95% CI of the bootstrap mean. The result mirrors the general inverted U-shaped pattern of WID over age reported in Figure 2b of the main text.

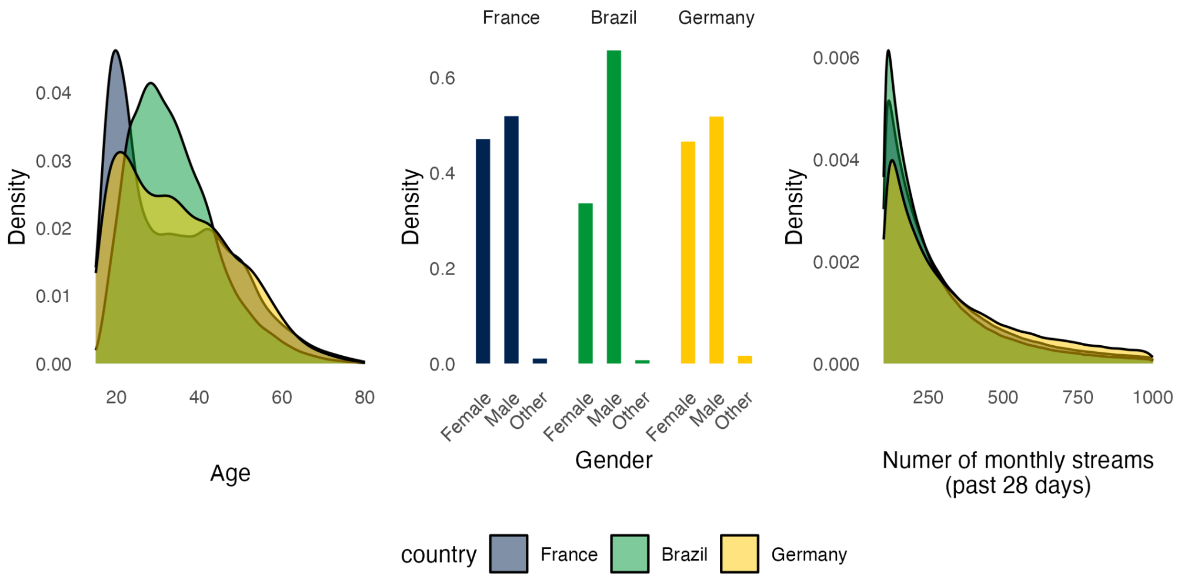

Fig. 9: Demography of Deezer users.

The demographics of the sampled users between the ages of 15 and 80. For gender reporting, those who have not self-identified as either 'Male' or 'Female' were grouped under 'Other' (for available gender selection options, see 'Age and gender' section in Methods). The number of monthly streams exhibited a heavy-tailed distribution and was capped at 1,000 streams for visualisation purposes.

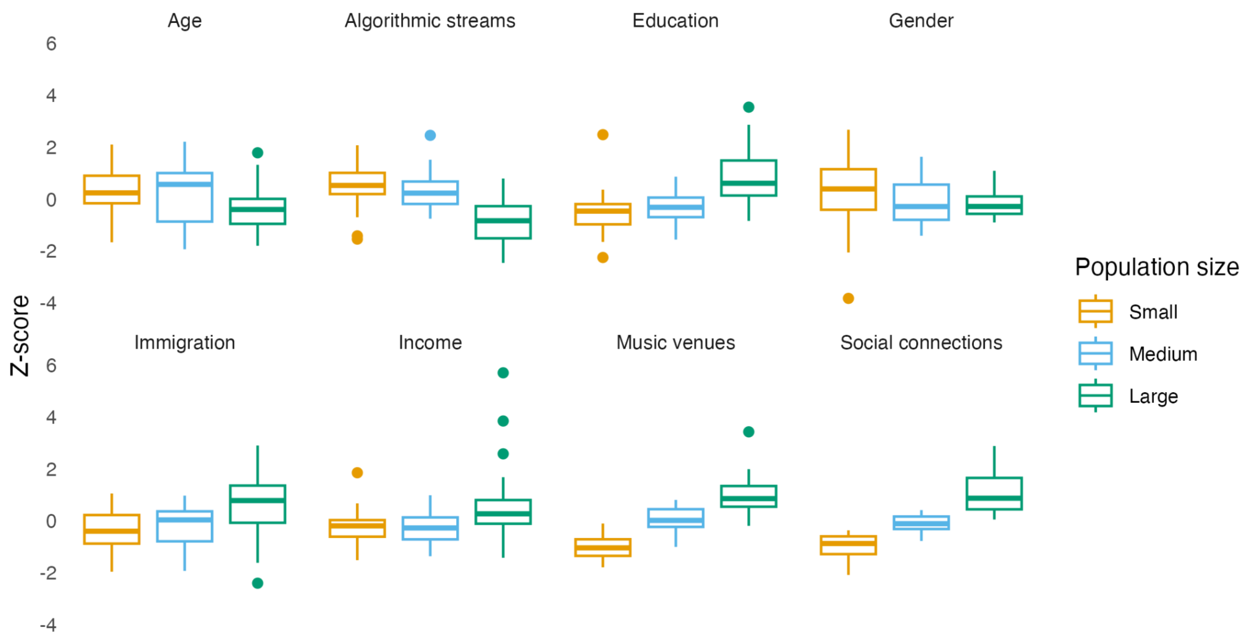

Fig. 10: Socio-demographic difference by population size.

Comparison of socio-demographic variables adopted in the DAG models. 96 French areas are divided into three quantile groups (small, medium, large; 32 areas each) based on population size. Each variable was normalised using Z-scores and then aggregated by the mean at the NUTS3 unit level. Box and whiskers show the median as centre, 25% and 75% quantile hinges, 1.5x interquartile range whiskers.

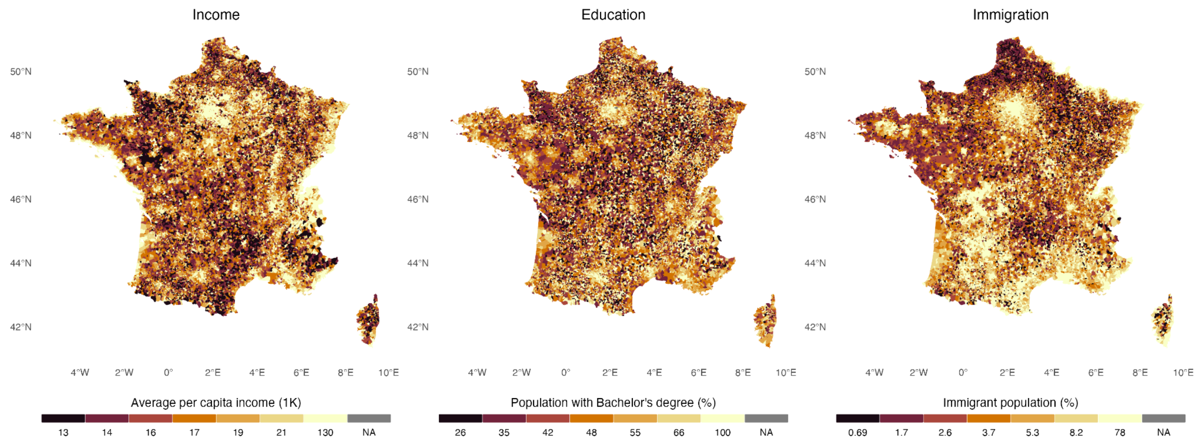

Fig. 11: Map of French communes by income, education, and immigration.

A map of France with 35,083 communes coloured by the log quantile categories. Income is derived from median GDP, education is the proportion of residents with a university Bachelor's degree, and immigration is the proportion of residents that do not hold French nationality. For more details, see ‘Immigration, education, and income’ section in Methods.

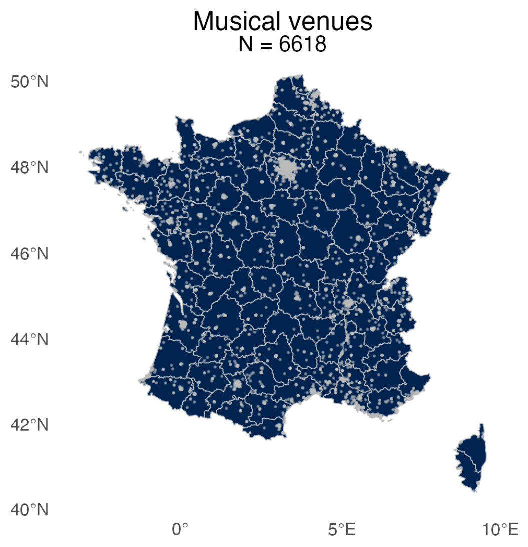

Fig. 12: Map of music venues in France.

A map of music venues derived from the Songkick database by their geocoordinates, collected across all venues existing through a search in August 2023.

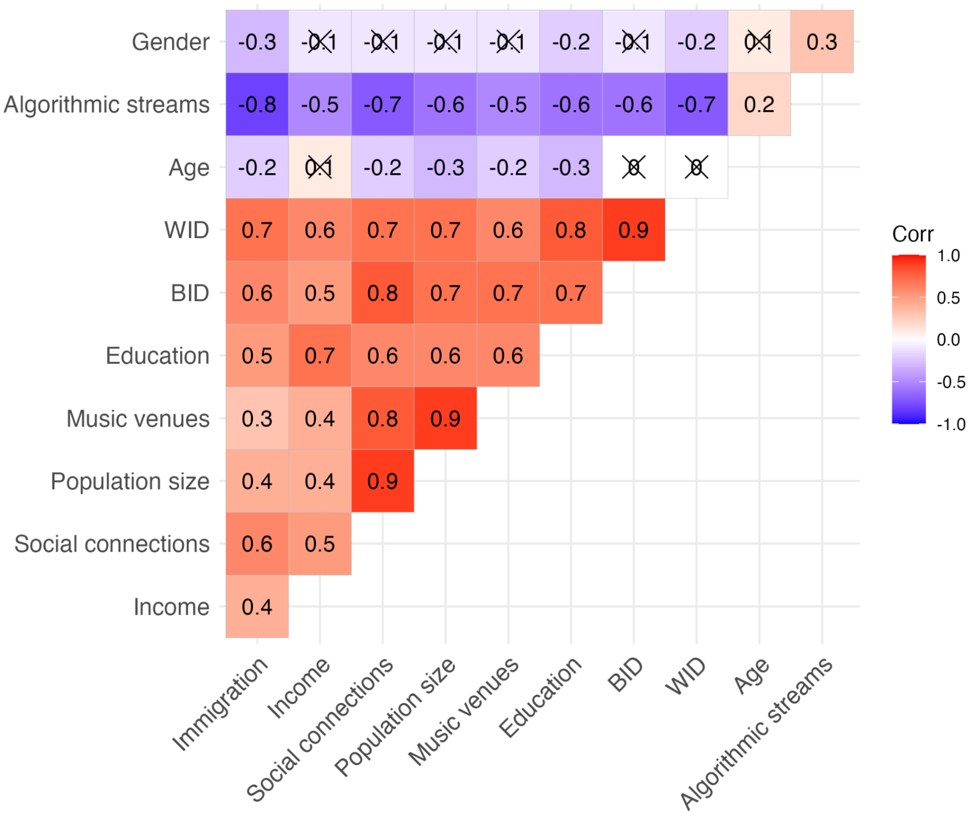

Fig. 13: DAG variables correlation matrix.

Two-tailed Pearson correlation matrix of DAG variables. Crossed boxes indicate no significance at P = 0.05 after adjusting for multiple comparisons (‘Statistical analysis’ in Methods). Each variable was aggregated by the mean at the NUTS3 unit level.

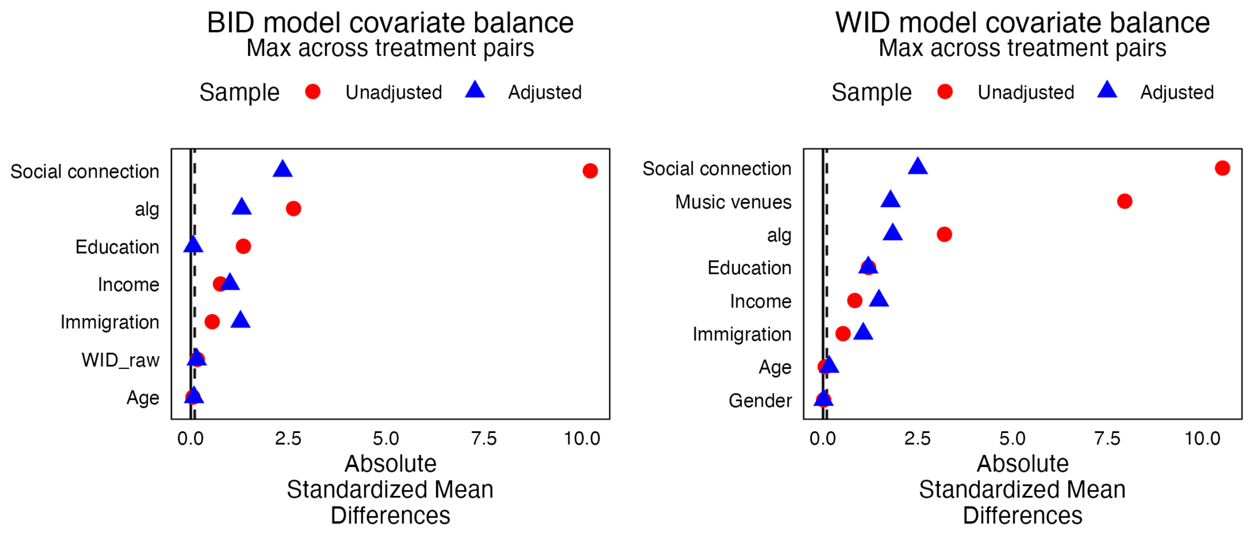

Fig. 14: Standardised mean differences.

Absolute standardised mean differences to compare the group balance after applying propensity score weights (blue triangle) and before adjustment (red circles), related to causal inference testing ('Causal inference' section in Methods). Higher values indicate larger maximum group-level differences across the treatment pairs. The closer the values are to 0 (dashed line), the better the balance across the groups (i.e., less differences).

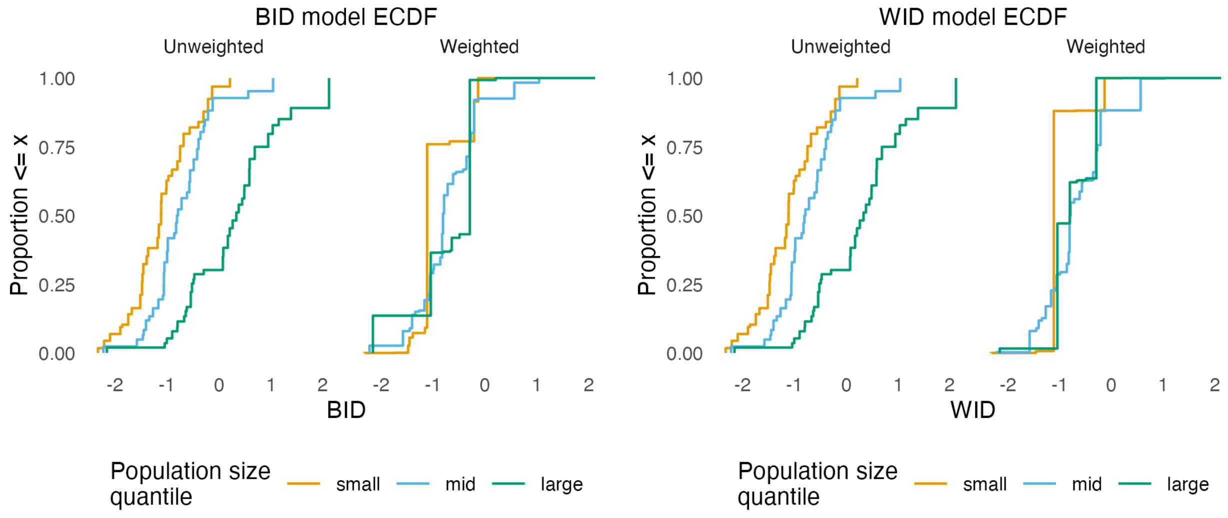

Fig. 15: ECDF distribution of weights.

Using empirical cumulative distribution function (ECDF) to compare balance across the groups without the weighting and with inverse propensity weights, related to causal inference testing (‘Causal inference’ section in Methods). When weighted, there is less gap between the population size quantile groups, suggesting there is a better balance across the covariates between the groups.

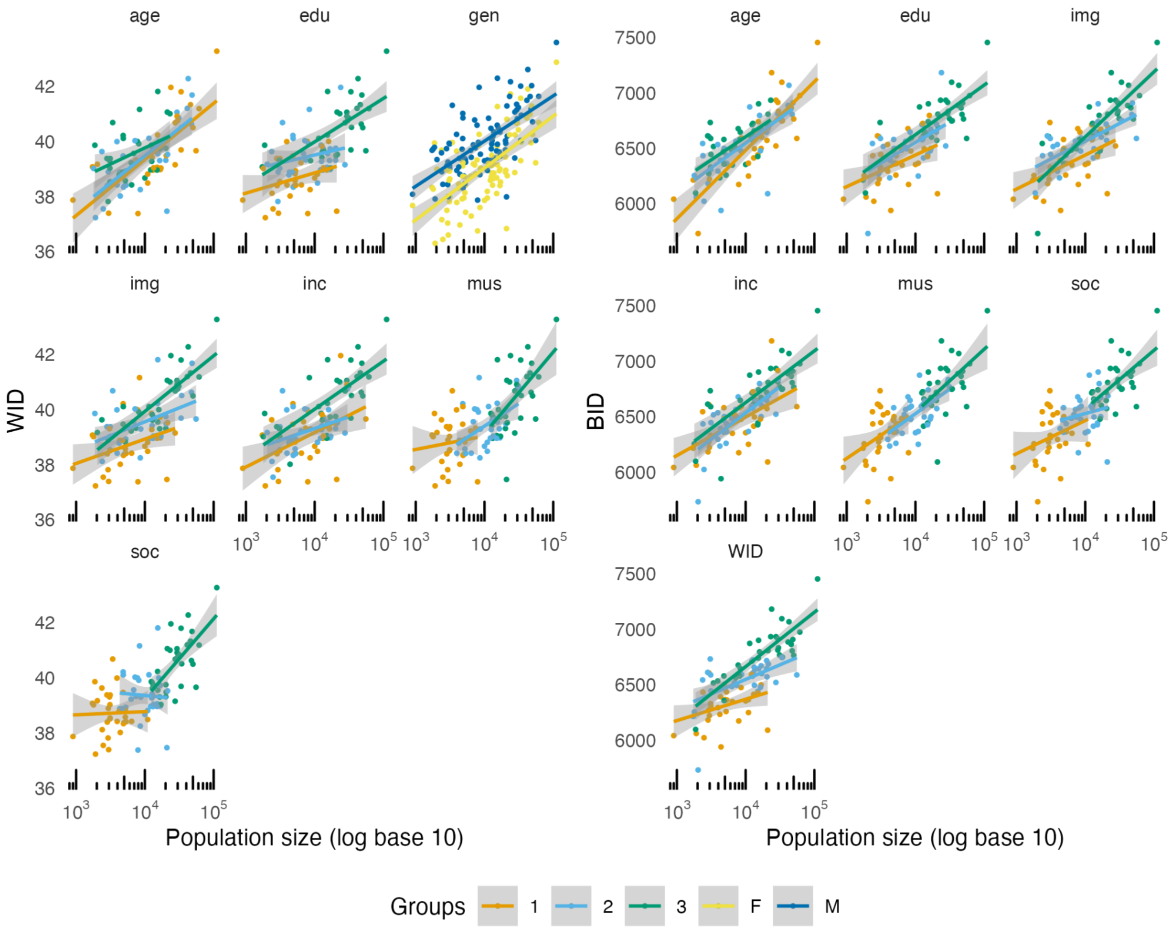

Fig. 16: Split quantile trends.

Relationship between population size and BID and WID using quantile splits of each confounder included in the DAG models. For instance, when stratifying the users by income (inc), regardless of their income quantile, the general association between the increase of both BID and WID with population size is consistently observed. The lines are GAM fitting and shaded areas represent 95% CI.

Supplementary Tables

Table 1: Pearson and Spearman correlations.

Pearson and Spearman correlations with BID and WID. BID is separately computed by grouping the streams in the units of songs, artists, and genres. WID is measured using the inverse of GS-Score. BID is measured using Hill's effective number with *q* order of 1 (i.e., Shannon entropy; 'Measuring diversity' section in Methods). All values are derived from bootstrapped means and brackets indicate 95% CI ('Statistical analysis' section in Methods). Note that the 'genre' grouping of BID reveals the weakest correlations. This may be due to genre tags being noisy. Genre tags are derived directly from the artist or the music labels themselves. Often a global genre tag of the artist can be uniformly applied across their songs, or fixed for the album — as such, individual song-level characteristics may get lost. Moreover, some may provide more specialised tags such as 'French rap', while others prefer to use more general tags like 'rap', where the two will be treated as unique categories when computing BID. These inconsistencies likely affect the results.

	**WID**		**BID**					
	**GS-Score**		**Song**		**Artist**		**Genre**	
	Pearson (r)	Spearman (ρ)	Pearson (r)	Spearman (ρ)	Pearson (r)	Spearman (ρ)	Pearson (r)	Spearman (ρ)
**France**	.65 [.52, .75]	.60 [.45, .72]	.73 [.62, .81]	.74 [.63, .82]	.58 [.43, .70]	.54 [.37, .67]	.41 [.23, .56]	.37 [.18, .54]
**Brazil**	.32 [.20, .44]	.31 [.18, .43]	.40 [.28, .51]	.39 [.27, .50]	.50 [.39, .59]	.48 [.37, .58]	.33 [.21, .44]	.34 [.21, .45]
**Germany**	.34 [.17, .49]	.28 [.09, .44]	.79 [.70, .85]	.75 [.66, .83]	.40 [.23, .54]	.38 [.20, .53]	.39 [.22, .54]	.42 [.25, .56]

Table 2: Organic and algorithmic streams.

Pearson correlations between population size and BID computed independently for organic-only, algorithmic-only, and the two combined (‘Algorithmic streams’ section in Methods). 1,000 unique streams were drawn randomly per area and per listen type (i.e., organic and algorithmic listens). Generally, regardless of the grouping, two-tailed Pearson correlations were similar and all significant at $P < 0.001$ after adjusting for multiple comparisons. All values are derived from bootstrapped means and brackets indicate 95% CI of the bootstrap mean (‘Statistical analysis’ section in Methods).

	**Song**			**Artist**			**Genre**		
	Organic	Algorithm	Combine	Organic	Algorithm	Combine	Organic	Algorithm	Combine
**France**	.73 [.62, .81]	.70 [.58, .79]	.75 [.64, .83]	.58 [.43, .70]	.63 [.49, .74]	.69 [.56, .78]	.41 [.23, .56]	.33 [.13, .49]	.42 [.24, .57]
**Brazil**	.40 [.28, .51]	.53 [.43, .62]	.48 [.37, .58]	.50 [.39, .59]	.63 [.54, .70]	.58 [.49, .66]	.33 [.21, .44]	.33 [.21, .44]	.36 [.24, .47]
**Germany**	.79 [.70, .85]	.73 [.64, .81]	.85 [.79, .90]	.40 [.23, .54]	.76 [.67, .83]	.65 [.52, .74]	.39 [.22, .54]	.42 [.26, .56]	.51 [.36, .63]

Table 3: Alternative diversity metrics of BID.

Different measures of BID with varying orders of *q* of Hills' number (also referred to as 'effective number of species'; 'Measuring diversity' section in Methods). Note, Gini-coefficient is in the inverse direction, where lower values indicate more uniformity in the distribution (i.e., more diversity). Additionally, two-tailed Pearson correlations between these diversity metrics and log population size are reported. All correlations with population size were significant at P < 0.001 after adjusting for multiple comparisons. All values are derived from bootstrapped means and brackets indicate 95% CI of the bootstrap mean ('Statistical analysis' section in Methods).

	**Hill's number (effective number)**			**Gini-coefficient**
	Richness q = 0	Shannon-entropy q = 1	Simpson q = 2	
**France**	955 [936, 971]	936 [907, 959]	904 [858, 940]	0.04 [0.03, 0.06]
**Brazil**	923 [870, 960]	886 [802, 942]	821 [681, 914]	0.07 [0.04, 0.12]
**Germany**	973 [957, 988]	962 [937, 984]	941 [896, 975]	0.03 [0.01, 0.04]
	**Correlations with population size** Pearson r			
**France**	.74 [.63, .82]	.73 [.62, .81]	.71 [.60, .80]	- .74 [-.82, -.63]
**Brazil**	.42 [.30, .52]	.40 [.28, .51]	.38 [.26, .48]	- .42 [-.52, -.30]
**Germany**	.80 [.72, .86]	.79 [.70, .85]	.76 [.67, .83]	- .80 [- .86, - .72]

Table 4. Spatial auto-correlation.

We applied several techniques to test for geographical spatial auto-correlation effects in our results. We report the standard measure of Moran's I, where values closer to 1 indicate more spatial clustering while closer to 0 indicate more random distribution across the geography. Except for BID in Germany, all were significant indicating the presence of spatial auto-correlations. Nevertheless, we note that our analysis focuses on the relationship between cultural diversity and population size, and proximity to an urban centre can be one of the reasons for diversity. In this respect, we view the existing spatial effects as a feature of our analysis rather than a confounding factor that needs to be controlled for. The phenomenon that large cities—and by extension, their neighbouring areas that are also usually large—exhibit more cultural diversity is a central aspect of our investigation.

To further test for spatial effects, we performed spatial detrending analysis by subtracting the mean values of neighbouring areas (excluding the focal area itself) from the dependent variable (either BID or WID) of the area. Neighbours were defined as areas within a 100km radius. This process ensures that spatial correlations are significantly reduced by removing the regional mean. After this adjustment, positive correlations persisted in all cases. We also tested whether the correlations of BID and WID still hold using a lower spatial resolution. In France and Germany, there exists NUTS2 units area data that is a higher level geographical boundary to NUTS3 used in our study. We thus tested for these two countries, while Brazil was excluded from this analysis due to not having equivalent multiresolution regional data. BID in both countries were significant and comparable to NUTS3 unit level correlations (Supplementary Table 1), while WID for Germany was insignificant ($P > 0.050$), two-tailed and correcting for multiple comparisons.

	**Moran's I**			
	**BID**		**WID**	
	Moran's I	P	Moran's I	P
**France**	0.50	< 0.001	0.50	< 0.001
**Brazil**	0.30	< 0.001	0.17	0.003
**Germany**	0.04	0.320	0.42	< 0.001
	**Spatial detrending**			
	**BID**		**WID**	
	Pearson r	P	Pearson r	P
**France**	.27 [.08, .45]	0.007	.37 [.18, .53]	0.001
**Brazil**	.60 [.51, .68]	< 0.001	.37 [.25, .48]	< 0.001
**Germany**	.75 [.66, .82]	< 0.001	.39 [.22, .54]	< 0.001
	**Lower spatial resolution (NUTS2 level)**			
	**BID**		**WID**	
	Pearson r	P	Pearson r	P
**France**	.82 [.60, .92]	< 0.001	.56 [.18, .79]	0.007
**Germany**	.45 [.13, .69]	0.008	.04 [-.30 .38]	0.812

Table 5. DAG variables.

Descriptive statistics of the variables included in the DAG model. INSEE = The National Institute of Statistics and Economic Studies of France. Granularity represents the level at which the information is used to proximate each user's attribute. When a variable did not follow a Gaussian distribution, they were log-transformed and indicated as Y, else used as raw values and indicated as N. The resulting distribution of each variable is visualised using histograms.

Variable	Source	Area Unit	Log Transform	Histogram
**Age**	Self-report	User	N	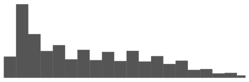
**Gender**	Self-report	User	N	
**Population Size**	Number of users	NUTS3	Y	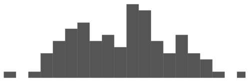
**Social Connections**	Facebook Social Connectedness Index (SCI)	NUTS3	Y	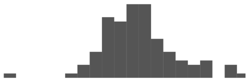
**Music Venues**	Number of music venues on SongKick	NUTS3	Y	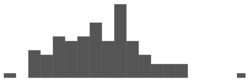
**Immigration**	INSEE	Municipalities	Y	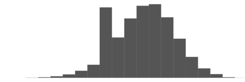
**Education**	INSEE	Municipalities	N	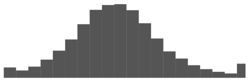
**Income**	INSEE	Municipalities	Y	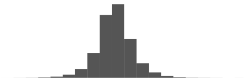
**Algorithm**	Computed over streams (N algorithmic stream / total N streams)	NUTS3	N	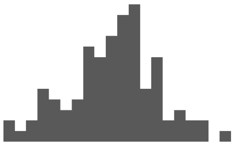
**WID**	Computed over users (GS-Score)	NUTS3	N	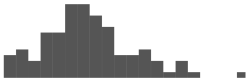
**BID**	Computed over streams (Hill's number $q = 1$)	NUTS3	Y	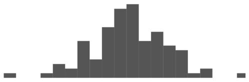

Table 6. France Facebook users demography comparison.

As a validation step for the 'social connection' measure, which we included as a confounder in the causal inference ('Causal inference' in Methods), we compared user demographics between Facebook and Deezer in France. We obtained demographic information for French Facebook users in March 2023 from a private marketing company (https://napoleoncat.com/stats/facebook-users-in-france/2023/03). We then used the age categories in this data to compare with our Deezer user sample.

	Facebook		Deezer	
**Age category**	**Male (%)**	**Female (%)**	**Male (%)**	**Female (%)**
13-17	1.5	1.7	3.5	5.6
18-24	10.0	10.4	16.0	15.8
25-34	12.2	12.4	9.3	11.6
35-44	8.9	9.8	8.2	9.8
45-54	6.7	7.6	5.8	7.8
55-64	4.3	5.6	1.8	3.0
65+	3.7	5.2	0.6	1.1

References

1. Ankan, A., Wortel, I. M. N. & Textor, J. Testing Graphical Causal Models Using the R Package “dagitty”. *Curr. Protoc.* **1**, e45 (2021).
2. Textor, J., Zander, B. van der & Ankan, A. dagitty: Graphical Analysis of Structural Causal Models. (2023).
3. Pearl, J., Glymour, M. & Jewell, N. P. *Causal Inference in Statistics: A Primer*. (John Wiley & Sons, 2016).
4. Cinelli, C., Forney, A. & Pearl, J. A Crash Course in Good and Bad Controls. *SSRN Electron. J.* (2020).
5. Rosenbaum, P. R. & Rubin, D. B. The Central Role of the Propensity Score in Observational Studies for Causal Effects. *Biometrika* **70**, 41–55 (1983).
6. Greifer, N. WeightIt: weighting for covariate balance in observational studies. *R Package Version 09 0* (2020).
7. Lee, B. K., Lessler, J. & Stuart, E. A. Weight trimming and propensity score weighting. *PloS One* **6**, e18174 (2011).
